# A Novel Optimized Hybrid Deep Learning Framework for Mental Stress Detection Using Electroencephalography

**DOI:** 10.3390/brainsci15080835

**Published:** 2025-08-04

**Authors:** Maithili Shailesh Andhare, T. Vijayan, B. Karthik, Shabana Urooj

**Affiliations:** 1 Department of Electronics Communication Engineering, Bharath Institute of Higher Education and Research, Chennai 600073, India; tvij16@gmail.com (T.V.);; 2 Department of Electronics and Telecommunication Engineering, Pimpri Chinchwad College of Engineering and Research Ravet, Pune 412101, India; 3 Department of Electrical Engineering, College of Engineering, Princess Nourah bint Abdulrahman University, P.O. Box 84428, Riyadh 11671, Saudi Arabia

**Keywords:** deep convolutional neural network, human behavior, deep belief network, deep learning, long short-term memory, stress detection

## Abstract

Mental stress is a psychological or emotional strain that typically occurs because of threatening, challenging, and overwhelming conditions and affects human behavior. Various factors, such as professional, environmental, and personal pressures, often trigger it. In recent years, various deep learning (DL)-based schemes using electroencephalograms (EEGs) have been proposed. However, the effectiveness of DL-based schemes is challenging because of the intricate DL structure, class imbalance problems, poor feature representation, low-frequency resolution problems, and complexity of multi-channel signal processing. This paper presents a novel hybrid DL framework, BDDNet, which combines a deep convolutional neural network (DCNN), bidirectional long short-term memory (BiLSTM), and deep belief network (DBN). BDDNet provides superior spectral–temporal feature depiction and better long-term dependency on the local and global features of EEGs. BDDNet accepts multiple EEG features (MEFs) that provide the spectral and time-domain features of EEGs. A novel improved crow search algorithm (ICSA) was presented for channel selection to minimize the computational complexity of multichannel stress detection. Further, the novel employee optimization algorithm (EOA) is utilized for the hyper-parameter optimization of hybrid BDDNet to enhance the training performance. The outcomes of the novel BDDNet were assessed using a public DEAP dataset. The BDDNet-ICSA offers improved recall of 97.6%, precision of 97.6%, F1-score of 97.6%, selectivity of 96.9%, negative predictive value NPV of 96.9%, and accuracy of 97.3% to traditional techniques.

## 1. Introduction

Mental stress is an inexorable issue faced by human beings irrespective of age, religion, ethnicity, region, and gender. Mental stress affects and limits an individual’s ability to disrupt daily routines [1]. In psychology, stress combines the perception of stress or a situation with the body’s response to it. Stress is generally triggered when an individual encounters adverse conditions, such as mental, physical, or emotional stressors. The stressors were grouped into internal and external. Internal stressors depend on individual perceptions, thoughts, and personalities. External stressors include relationship problems; financial difficulties; work pressure; and professional, political, and religious pressures [2]. Stressors include mental arithmetic tests, picture perception tests, and rapidly changing tasks. Physical stressors included exercise, physical activity, painful stimuli, or sleep deprivation. Emotional stressors include videos or songs [3].

Mental stress is classified as either chronic or acute. Acute stress occurs when an individual is exposed to short-duration stressors such as public speaking or job interviews. Long-term and frequent exposure to stressors, such as poor sleep habits, stressful jobs, and poor relationships, lead to chronic stress. Various physiological changes occur in an individual’s body to deal with stress [4]. Stress may cause the release of cortisol, noradrenaline, and adrenaline, thereby providing instant energy to the body. Afterwards, the parasympathetic nervous system regulates the body to normal conditions (homeostasis condition) without any significant harm to the body. Continuous or long-term exposure to stress affects an individual’s mental and physical health. Stress leads to distinct health issues such as stroke, hypertension, cardiac arrest, coronary artery disease, persistent pain, anxiety, muscle exhaustion, and depression [5].

Psychiatrists and clinicians analyze stress using self-report questionnaires. Various questionnaires are used to analyze stress, such as the daily stress inventory, perceived stress scale, and relative stress scale. However, their trust and efficiency were highly subjective and prone to incorrect or invalid answers. Questionnaire-based stress analysis has a high error rate owing to social response and desirability biases. Additionally, behavior analysis based on vocal and non-verbal indications (rapid eye movement and body gesture) and visual responses was utilized for stress analysis [6]. However, behavioral analysis can vary in conscious states. The questionnaire reports and behavioral analysis are subject to erroneous expert knowledge due to fatigue, inadequate expert knowledge, tiredness, and bias. Stress refers to the physiological changes in a person affected by the autonomic nervous system. These changes include physiological modalities, such as eye gaze, skin temperature, pupil diameter, voice blood volume pressure, heart rate variability (HRV), and electrodermal conductance. However, physiological signals are significantly affected by environmental conditions and health. Skin diseases and environmental parameters, such as temperature and humidity, strongly influence electrodermal conductance [7].

Researchers have recently focused on various neuro-signals and neuroimaging techniques for stress analyses. These modalities include EEG, near-infrared spectroscopy, positron emission tomography, and functional magnetic resonance imaging [8]. EEG has shown greater reliability, robustness, and accuracy than neuroimaging techniques. EEG-based stress analysis is inexpensive and offers a high temporal resolution [9]. EEG is a noninvasive technique that captures oscillations produced by electrical brain activity by mounting electrodes over the scalp. EEG signals have amplitudes up to 200 V. EEG has different frequency bands, which represent distinct mental states such as delta (0.5–4 Hz), theta (4–8 Hz), alpha (8–13 Hz), beta (13–30 Hz), and gamma (>30 Hz). Details of the EEG signals are provided in Table 1.

AI-based stress detection systems are categorized into machine learning and deep learning-based stress detection systems. Traditional ML-based systems involve preprocessing, feature extraction, and classification. Preprocessing consisted of signal standardization, normalization, noise removal, artifact removal, data augmentation, signal cropping, and appending. It is essential to enhance the quality of the EEG signals. EEG signals often suffer from artifacts generated from body muscles, ocular signals, and body movements. These artifacts degrade EEG quality and lead to poor feature representation.

The next phase includes feature extraction, which acquires the unique characteristics of the EEG using computational algorithms. These features are important for segregating normal EEGs from stressed EEGs. ML algorithms are highly reliant on the data used for training. Quantity, diversity, and quality affect the performance of ML algorithms. Biased and insufficient data can lead to inaccurate predictions. ML algorithms are less suitable for larger datasets and require more learning time. The performance of the ML classifier depends largely on its features. Redundancy and non-distinct features reduce the model’s performance. The ML algorithm must provide better results with limited data. ML models require more contextual understanding and may provide less accuracy. Feature extraction algorithm selection is challenging owing to the unavailability of standard benchmarks for algorithm selection. The ML models show interior feature representation capabilities. Uneven training samples lead to a class-imbalance problem. The ML algorithm provides overfitting and shows a lower generalization capability for newer data. The outcomes of ML algorithms are easily affected by noise and signal artifacts. Traditional systems suffer from class imbalance problems owing to uneven training dataset samples. Generating a dataset for the stress class is challenging because of the micro-nature of the stress and reliability issues of the stressor [10].

This study presents a novel hybrid BDDNet for stress detection using EEG signals. The main contributions of this study are summarized as follows.

- An efficient channel selection scheme using a novel improved CSA algorithm to select distinctive channels and reduce the computational complexity of the system.
- Stress representation using Multiple EEG features (MEFs) provides time and frequency domain features.
- Implementation of a novel BDDNet for stress detection where DCNN provides the spatial and spectral domain features of EEG; BiLSTM offers the temporal and long-term dependency of the EEG and DBN to provide multilevel hierarchical features of the EEG.
- Hyper-parameter optimization of the BDDNet using novel EOA to boost training performance.

The remainder of this paper is structured as follows. Section 2 provides a literature survey of recent stress detection schemes. Section 3 describes the overview of the proposed methodology in detail. Section 4 offers the details about the proposed BDDNet. Section 5 describes the implementation details of EOA-based hyper-parameter optimization. Further, Section 6 presents the experimental results and discusses the analytical findings. Section 7 presents the conclusions, imperative findings, and directions for future work.

## 2. Related Work

Various deep learning-based schemes have been proposed in recent years to enhance the performance of stress-detection schemes. Roy et al. [11] presented CBGG, which is a hybrid combination of CNN, BiLSTM, and two gated recurrent unit GRU layers for EEG-based stress detection. It uses a discrete wavelet transform (DWT) representation to describe the spectral and temporal characteristics of EEGs. DWT minimizes the nonlinearity and non-stationarity of EEGs. It offers an overall accuracy of 98.1% for the simultaneous task EEG workload (STEW) dataset that includes 14-channel EEG signals. However, the effectiveness of stress detection is limited because of its high network complexity, higher recognition time, and extensive hyper-parameter tuning. In addition, selecting a DL algorithm to construct a hybrid classifier is a challenging task. Mane et al. [12] explored the amalgamation of 2D-CNN and LSTM for stress detection, which considered azimuthal projected images of alpha, theta, and beta signals as the input. The combination of CNN and LSTM assists in boosting the spectral and temporal depictions of the EEGs. This resulted in an overall stress detection rate of 97.8% for DEAP, 94.5% SEED, and 97.8% for (DEAP+SEED). The system required a higher training time of 4.2 h and a recognition time of 12.5 s. Patel et al. [13] investigated 1-D CNN and BiLSTM to enrich the spectral–temporal characteristics of EEGs. The stress detection model accepts time–frequency features to learn the local and global representations of the EEGs. It provides 88.03% accuracy for the DEAP dataset, but suffers from poor feature depiction and class imbalance problems.

Furthermore, Bhatnagar et al. [14] provided an EEGNet based on CNN, which accepts the mother wavelet decomposition of the EEG into five spectral bands for stress detection. It offered 99.45% accuracy for the in-house dataset created by capturing the EEGs while playing low- to high-pitched music. However, the dataset variability was limited owing to the limited sample size (45 subjects aged 13–21). According to Hafeez et al. [15], timing has a significant influence on stress. Researchers have observed that stress levels are greater for untimed tests than for timed tests based on real-time experimental data. Overall accuracy for the EEG signals in picture format was 70.67% and 90.46% for the LSTM and DCNN, respectively. The DCNN offers improved spatial correlation and connectivity among the various EEG bands. However, the temporal description of the signal and long-term reliance were absent from the 2D picture representation of the EEG. Geetha et al. [16] investigated an enhanced multilayer perceptron (EMLP) to identify stress by utilizing sleep patterns in EEG data. Owing to the extreme complexity of sleep patterns, the ability of the EMLP to extract complicated sleep pattern information from EEG signals is restricted for real-time analysis.

To identify epileptic seizures caused by stress and worry, Palanisamy et al. [17] used fuzzy c-mean (FCM) features, and LSTM was adjusted using a particle swarm algorithm (LSTM-PSO). The Hjorth Activity, variance, skewness, kurtosis, standard deviation, Shannon entropy, and mean are among the FCM properties. Location and random data augmentation resolve the class imbalance issue, creating synthetic EEG samples. It achieved 97% PSO-LSTM-based stress identification for the BONN EEG dataset, and an overall accuracy of 98.5% for FCM-PSO-LSTM. According to Bakare et al. [18], valence and arousal can provide stress information from different EEGs. KNN obtained better results for the smaller dataset, whereas the larger dataset did not provide encouraging results. Recurrent neural networks (RNNs) and random forests (RFs) were proposed by Khan et al. [19] for cross-dataset mental stress detection to improve the capacity of generalization of the stress detection scheme. It tests employing RNN and RF on the Game Emotion (GAMEEMO) dataset and the SJTU Emotion EEG Dataset (SEED) dataset for training. Regarding accuracy, the RNN performed better than the RF (83% for arousal and 75% for valence), with 87% for arousal and 83% for valence. Gonzalez-Vazquez et al.’s [20] proposal, which uses an 8-channel EEG for multilevel stress detection in serious gaming tasks, calls for gated recurrent units (GRUs). It performed well in stress detection with 94% accuracy, but its weak generalization limits its usefulness. Naren et al. [21] investigated a 1D CNN and Doppler characteristics for stress detection. The initial component of the 1D CNN was trained using induced stress via mirror tasks, the Stroop test, and arithmetic tests. Features of low, medium, and high stress levels were used to train the second portion of the 1D CNN. The SAM-40 dataset yielded an overall accuracy of 95.25 percent.

From an extensive survey of various stress-detection techniques, the following gaps were identified:

- Lower feature depictions of single-channel EEGs, low-frequency resolution issues, limited spectral–temporal representation, and inferior long-term dependency on EEG signals [22].
- The class imbalance problem provides the disparity between the qualitative and quantitative stress attributes of EEGs [23].
- Low accuracy for low arousal and valence EEG signals.
- Stress detection systems suffer from a low generalization capability, which limits their effectiveness in real-time implementation. DL-based systems have provided better results than ML-based stress-detection techniques [24].
- DL algorithms work as black boxes and have higher abstraction levels, failing to justify different features adequately. Thus, their explainability and interpretability were inferior.

## 3. Methodology

**Figure 1:**
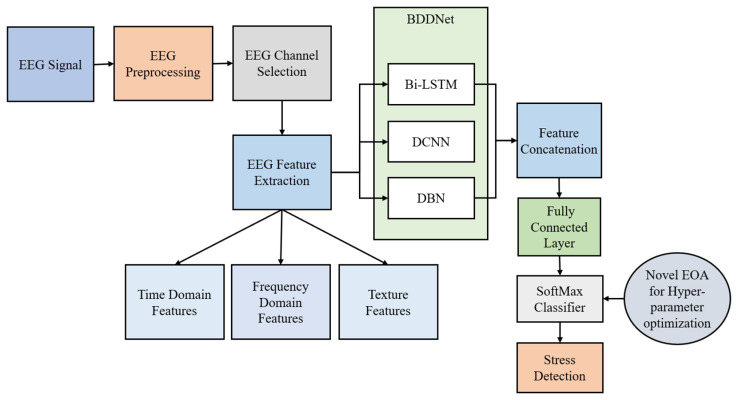
Flow diagram of the proposed stress detection system.

Figure 1 shows a flow diagram of the proposed stress-detection framework, which encompasses EEG preprocessing, channel selection, feature extraction, and stress detection using a novel DL framework.

### 3.1. EEG Preprocessing

EEGs often affect noise and artifacts due to electrooculogram (EOG), electromyogram (EMG), and electrocardiogram (EEG) signals, thereby reducing stress. Minimizing noise and artifacts is essential for enhancing the EEG quality and improving the mental stress detection performance. The EEG was passed through a finite impulse response (FIR) filter with bands between 0.75 Hz and 45 Hz to minimize noise. Furthermore, a wavelet packet transform-based soft thresholding scheme was used for EEG denoising, which minimized the noise and artifacts in the EEG signal without degrading the actual information in the EEG [25]. The EEG signals were decomposed into three levels using a Daubechies filter (db3). The decomposed packets are compared with Donoho’s soft thresholding value and reconstructed to attain an enhanced signal.

### 3.2. Channel Selection Using Improved Crow Search Algorithm

Crows are regarded as the most intelligent bird species. They stow away food and retrieve it when needed. They stick close to one another, watch and investigate where other crows keep their food, and then take it after the owner has gone [26]. Crows will change their hiding spot if they believe that they are being followed to protect their food from being taken. The algorithm comprises four fundamental principles, all of which are derived from the behavioral patterns of crows.

- Crows tend to congregate in large numbers.
- Crows have excellent memories and can recall exactly where food was hidden.
- Crows are known to stick together in order to steal food.
- Crows have the capacity to perceive their environment. When they become aware that they are being followed, they move the food they have hidden to protect it from being taken.

**Figure 2:**
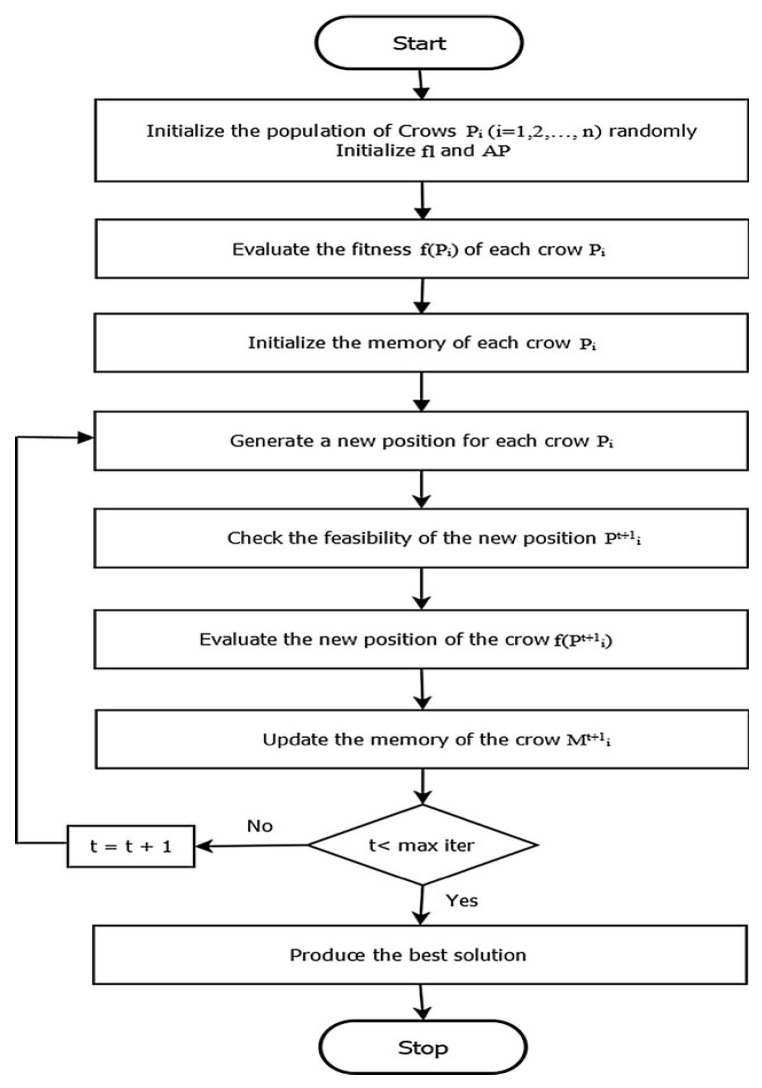
Flowchart of novel ICSA for EEG channel selection.

The Crow Search Algorithm (CSA) is an innovative type of swarm intelligence optimization algorithm that was developed by modeling the intelligent actions that crows perform while searching for and locating food. The method is characterized by its straightforward structure, limited number of control parameters, and straightforward applications. The fact that only two factors can be adjusted makes it straightforward, which in turn makes it extremely appealing for use in a variety of technical domains [27]. The traditional CSA algorithm provides a poor optimization solution because of its low solution diversity, poor exploration and exploitation, and inferior optimization results [28]. The proposed improved CSA uses elite learning with spiralized learning and a weak member replacement scheme to enhance the solution diversity, convergence, and balance between exploration and exploitation. The flow of the proposed ICSA-based channel selection process is illustrated in Figure 2.

- **Process of ICSA**

The crow search algorithm (CSA) imitates the behavior of crows by storing excess food and recovering it when required. According to optimization theory, the crow acts as the searcher, the environment around it serves as the search space, and storing the location of food in a manner that is entirely at random is a possible option. The CSA adheres to the following ideals, which are derived from the lifestyle of crows: (1) crows are gregarious creatures; (2) crows are able to recall the position of concealed food; (3) crows will follow each other and take food from each other; and (4) crows try their utmost to prevent other crows from stealing their food. The algorithm for the CSA is as follows [26,27,28]:

**Step 1:** *Initialize the problem statement and algorithm parameters.*

*N: Flock size*
*Ft: Flight length*
*Iter_max: Maximum iterations*
*AP: Awareness probability*

**Step 2:** *Initialize the crow position and memory.*

The flock is composed of N crows that are distributed randomly over a d-dimensional search space, where d denotes the total number of possible channels. The initial row positions are represented by Equation (1):

(1) $$crows=\left[\begin{array}{cccc} x_{1}^{1} & x_{2}^{1} & \ldots & x_{d}^{1}\\ x_{1}^{2} & x_{2}^{2} & \ldots & x_{d}^{2}\\ \vdots & \vdots & \vdots & \vdots \\ x_{1}^{N} & x_{2}^{N} & \ldots & x_{d}^{N} \end{array}\right]$$

Each crow had initialized memory. It is supposed that crows have hidden their food in their first placements because they are thought to have little experience at this point. The memory of crows is described by Equation (2).

(2) $$crows=\left[\begin{array}{cccc} m_{1}^{1} & m_{2}^{1} & \ldots & m_{d}^{1}\\ m_{1}^{2} & m_{2}^{2} & \ldots & m_{d}^{2}\\ \vdots & \vdots & \vdots & \vdots \\ m_{1}^{N} & m_{2}^{N} & \ldots & m_{d}^{N} \end{array}\right]$$

**Step 3:** *Evaluate the fitness of each crow.*

By entering the values of the decision variables into the objective function for each crow, the quality of its location was calculated. The objective function for channel selection considers the entropy (EN) and covariance (CV) of the channels, which helps to select salient channels with higher information. Channel selection assists in minimizing the computational effort of the stress detection system. The objective function utilized for computing the fitness is provided in Equation (3). Here, $w_{1}$ and $w_{2}$ were selected such that $w_{1}+w_{2}=1$.

(3) $$fitness=w_{1}*EN+w_{2}*CV$$

**Step 4:** *Generate new crow position.*

To update, the crow selects a flock member at random, such as Crow J, and follows it to find the location of concealed food. In Equation (4), the new location of the crow is updated.

(4) $$x^{i,iter+1}=\left\{\begin{array}{c} x^{i,iter}+r_{i}\times {fl}^{iter}\times (m^{j,iter}-x^{i,iter})\;for\;r_{j}\geq {AP}^{j,iter}\\ \;A\;random\;number\;otherwise \end{array}\right.$$

The traditional CSA updates the population randomly, leading to poor solution diversity and convergence. However, updating the best and worst solutions is neglected, which creates a poor balance between exploration and exploitation. Thus, the improved CSA provides two competitive learning schemes to enhance the diversity of solutions: convergence and exploration–exploitation search space. The LFEL strategy updates the best object using the Levy step function to improve the exploration space of the algorithm. It considers the first two solutions ($x_{best1}\;and\;x_{best2})$ with the highest fitness values, as given in Equation (5).

(5) $$x_{i}^{LFEL}=x_{best1}+\left(2*r1-1\right)*levy(\beta )*(x_{best1}-x_{best2})$$

Here, $x_{i}^{LFEL}$ indicates the updated object obtained using the LFEL scheme, β denotes the distribution index, and r1 is an arbitrary index between 0 and 1.

Furthermore, it uses the RWM strategy to boost exploitation of the algorithm. Every weak solution is updated towards the best solution to enhance the exploitation search space of the CSA. The crow position is updated using the RWM strategy, as shown in Equation (6).

(6) $$x_{i}^{RWM}=x_{worst}+r2*(x_{best}-x_{worst})$$

Here, $x_{i}^{RWM}$ signifies the updated crow using the RWM scheme, r2 is a random number between 0 and 1, and $x_{worst}$ stands for a solution with the worst fitness.

**Step 5:** *Feasibility checking of new crow positions*.

The new position in each crow was examined for viability. A crow changes position if its new location is viable. Otherwise, the crow does not go to the new spot and remains in its existing location.

**Step 6:** *Compute the fitness value for newer position*.

**Step 7:** *Update the crow memory using Equation (7).*

(7) $$m^{i,iter+1}=\left\{\begin{array}{c} m^{i,iter+1}\;if\;fitness\;of\;m^{i,iter+1}>fitness\;of\;m^{i,iter}\\ \;m^{i,iter}\;otherwise \end{array}\right.$$

**Step 8:** *Check the termination criteria*. Steps 4–7 were repeated until iter_max was achieved. After the termination requirement is satisfied, the optimal memory position relative to the value of the objective function is provided as a solution to the optimization problem. The algorithm for the ICSA is given as follows (Algorithm 1):
**Algorithm 1:** ICSA for EEG Channel Selection**Input:** Random channel population
**Output:** Optimized ChannelsStep 1:Initialize the problem and parameters. 
Set flock size N, flight length Ft, maximum iterations Iter_max, and awareness probability AP. Step 2:Initialize crow positions and memory.    i.Randomly generate initial positions of N crows in a d-dimensional search space, as given in Equation (1).    ii.Initialize memory assuming each crow hides food at its initial location, as given in Equation (2). 
Step 3:Evaluate initial fitness. 
- Compute fitness of each crow using the objective function considering entropy (EN) and covariance (CV), as given in Equation (3). Step 4:Generate new crow positions. 
For each crow i:    i.Randomly choose crow j.    ii.Update position based on Equation (4).    iii.Apply the LFEL strategy to update the best solutions using Equation (5).    iv.Apply the RWM strategy to guide weaker solutions using Equation (6). 
Step 5:Check feasibility of new positions. 
- If the new position is feasible, update the crow’s position. Otherwise, retain the old position. Step 6:Recalculate fitness for updated positions. Step 7:Update crow memory. 
i. Compare current and previous fitness values. 
ii. Update memory as per Equation (7). Step 8:Termination check. 
i. Repeat Steps 4–7 until Iter_max is reached. 
ii. Return the best memory position as the optimal solution. 

### 3.3. Multiple EEG Features

The features were classified into time-domain, spectral, and textural features of the EEG.

**A.**  **Time-Domain EEG Features**

- **Mean, Standard Deviation, and Variance**

The mean and SD offer time-domain variations in the EEG owing to stress. The variance provides consistency in the EEG patterns. The mean (μ), standard deviation (σ), and variance (*vr*) for EEG signal *E* having *N* samples are depicted in Equations (8)–(10), respectively.

(8) $$\mu =\frac{1}{N}\sum_{i=1}^{N}E_{i}$$

(9) $$\sigma =\sqrt{\frac{1}{N}\sum_{i=1}^{N}{\left(E_{i}-\mu \right)}^{2}}$$

(10) $$vr=\frac{1}{N}\sum_{i=1}^{N}{\left(E_{i}-\mu \right)}^{2}$$

- **Hjorth’s Parameters**

Hjorth’s parameters provide the activity, mobility, and complexity of the EEG signal. The EEG signal activity depicts the signal’s variance over time [8]. Higher activity due to stress in the brain indicates a higher activity value compared with a normal mental state. Mobility is the square root of the ratio of the variance of the first-order derivative of EEG to that of EEG over time. The activity (act or $\sigma^{2}$) is denoted by Equations (11) and (12), where ${eeg}_{i}$ denotes individual samples of EEG signals, $\bar{eeg}$ describes the mean of the EEG, and N signifies the total samples of the EEG. Mobility (mob) describes the frequency variations in the EEG, as given in Equation (13). Higher mobility indicates rapid variations in the EEG, representing higher brain activity or stress. A higher complexity (cmp) value represents more complex variations in brain activity that depict higher stress. The complexity is defined as the square root of the ratio of the mobility of the first derivative of EEG to the mobility of EEG, as given in Equation (14).

(11) $$act=\sigma^{2}=\frac{1}{N-1}\sum_{i=1}^{N}{\left({eeg}_{i}-\bar{eeg}\right)}^{2}$$

(12) $$\bar{eeg}=\frac{1}{N}\sum_{i=1}^{N}{EEG}_{i}$$

(13) $$mob=\sqrt{\frac{act\left(\frac{d\left(eeg\right)}{dt}\right)}{act(eeg)}}$$

(14) $$cmp=\sqrt{\frac{mob\left(\frac{d\left(eeg\right)}{dt}\right)}{mob(eeg)}}$$

- **Median**

The median value offers the central tendency of the EEG, which depicts the independence of the outliers.

- **ZCR**

ZCR offers randomness and noise in the EEG, which has a higher stress value. Stress causes instability in EEG patterns and provides larger transitions in the EEG. ZCR is computed using Equation (15), where **1**{.} provides one value as a sign that the current samples have changed from the previous samples that depict zero crossing. The signs of the EEG samples were obtained using sign function.

(15) $$ZCR=\frac{1}{N-1}\sum_{i=1}^{N-1}1\{sign(x[n])/sign(x[n-1])\}$$

- **RMS**

The RMS describes the overall signal power, and the entropy depicts random or irregular EEG patterns. The RMS value was computed using Equation (16).

(16) $$RMS=\frac{1}{N}\sqrt{\sum_{i=1}^{N}{E(i)}^{2}}$$

- **Line length (LL)**

The line length provides the overall vertical or curve length of the EEG signal, which shows the stress pattern in the signal. Equation (17) is used to calculate LL.

(17) $$LL=\sum_{i=1}^{N}(E\left(i\right)-E(i-1))$$

- **Shannon Entropy (SnE)**

Equation (18) is used to determine the uncertainty value in the EEG signal provided by SnE, where $p_{i}$ is the frequency of each sample in the EEG.

(18) $$SnE=-p_{i}\log p_{i}$$

- **Nonlinear Energy (NE)**

NE offers information regarding the irregular and non-linear patterns of EEG. The amplitude and oscillation shifted frequency values increased with the NE value. Equation (19) was used to calculate the NE.

(19) $$NE=\sum_{i=1}^{N-2}(E^{2}\left(i\right)-E\left(i+1\right)*E(i-1))$$

**B.**  **Frequency Domain Feature**

- **WPT**

WPT provides stationary and transient EEG patterns in the time–frequency domain. The EEG signal is split into five levels using a ‘db2’ filter that provides subbands. The fifth-level decomposition provided 32 subbands. Seven statistical features (mean, median, energy, skewness, kurtosis, variance, and entropy) were computed for each sub-band. The five-level decomposition provided 224 WPT features.

- **Energy (EN)**

Energy provides the EEG strength in distinct frequency bands. It depicts the transition from the normal state to stress and is computed using Equation (20).

(20) $$EN=\sum_{i=1}^{N}{\left[E\left(i\right)\right]}^{2}$$

- **Instantaneous Wavelet Moment of Frequency (IWMF)**

The IWMF offers dynamic disparities in EEG that describe the microarousal due to stress. The IWMF was estimated using Equation (21), where *E*[*k*] is the normalized PSD at frequency *f*[*k*].

(21) $$IWMF=\sum_{k}E\left[k\right]\cdot f(k)$$

- **Instantaneous Wavelet Bandwidth of Frequency (IWBF)**

The IWBF provides the bandwidth of the stress levels, as given in Equation (22). It is vital to discriminate between activities in the brain due to stress. PSD offers a high value for normal activity.

(22) $$IWBF=\sqrt{\sum_{k}{E\left[k\right]\cdot (f\left(k\right)-IMWF)}^{2}}$$

- **Spectral Kurtosis**

SK describes the non-Gaussian nature of the EEG pattern, which depicts complex EEG patterns.

**C.**  **Texture Feature**

Local temporal changes in the EEG signal pattern are provided by the local binary pattern (LBP) characteristics. Smaller amplitude fluctuations, micro-arousals, and transitory changes across the EEG due to stress may be obtained using the Local Neighborhood Difference Pattern (LNDP). The Local Gradient Pattern (LGP) offers directional changes in EEG to show prominent and subtle differences across complicated EEG patterns. Fluctuations in the EEG gradients (G), as shown in the equation, are provided by the LGP, where c signifies the center value in the window and $x_{i}$ represents the neighboring samples. LBP, LGP, and function f are given by Equations (23), (24), and (25), respectively. The details of all 527 EEG features for each channel are listed in Table 2.

(23) $$LBP\left(c\right)=\sum_{i=1}^{N}f(E\left(x_{i}\right)-E\left(c\right))*2^{i-1}$$

(24) $$LGP\left(c\right)=\sum_{i=1}^{N}f(G\left(x_{i}\right)-G\left(c\right))$$

(25) $$f\left(x\right)=\left\{\begin{array}{c} 1,\;x<0\\ 0,\;x\geq 0 \end{array}\right.$$

## 4. BDDNet for Stress Detection

The proposed BDD network combines Bi-LSTM, DCNN, and DBN to enhance feature representation. Bi-LSTM provides bidirectional long-term dependencies and a superior temporal representation of EEG features. The DCNN provides spatial detection and hierarchical abstract-level features that offer correlation and connectivity between the distinct local and global features of the EEG for stress detection. The DBN offers multilevel hierarchical features and representation of the complex patterns of the EEG signal for stress detection.

**A.**  ***Deep Convolution Neural Network***

CNNs have shown robustness in various signal processing applications. They are capable of learning EEG features independently. CNNs adaptively and automatically learn the hierarchical and spatial features of EEG signals using back-propagation learning. CNNs have the potential for various pattern recognition applications for biomedical image signals, audio, and time-series data [29,30,31,32]. The convolution layer is the chief building block of CNNs. This provides a hierarchical correlation between the different local and global characteristics of the EEG signal. The convolution layer provides local correlations and connectivity in EEG features. It offers hierarchical abstract-level EEG features that describe distinctive features depicting variations in the EEG signal. In this layer, the input signal is convolved with multiple convolution kernels to provide deep features, as expressed in Equations (26) and (27).

(26) $$E_{conv}\left(x,y\right)=EEG*K$$

(27) $$E_{conv}\left(x,y\right)=\sum_{i=1}^{R}\sum_{j=1}^{C}EEG\left(i,j\right)\cdot K(x-i,y-j)$$

Here, EEG denotes the original EEG signal, K denotes the convolution filter, and $E_{conv}$ denotes the convolution output.

Batch normalization converts the deep features into a normalized format to minimize outliers. This assists in accelerating the training performance of the DCNN. The BN operation for a batch size b is given by Equation (28). Here, $\mu_{b}$ and $\sigma_{b}$ denote the mean and variance over batch b, respectively, and ∝ and β indicates the scale and offset, respectively.

(28) $$BN\left(x\right)=\propto \cdot \frac{E_{conv}\left(x\right)-\mu_{b}}{\sigma_{b}}+\beta$$

The ReLU layer enhances the non-linear characteristics of the features by replacing the negative values with zero. This helps to improve the classification accuracy and lessen the vanishing gradient problem. The output RL of the ReLu layer is described by Equation (29).

(29) RL=max (0,BN(x))

The maximum pooling layer selects the maximum value from a local window. It chooses salient features and neglects non-salient or redundant features. Maximum pooling minimizes the feature dimensions and thus helps reduce the network’s trainable parameters.

The output of the last max pool layer was flattened and converted into a 1-D vector. The flattened vector is provided to the FCL, which links every neuron of one layer with all other neurons of the other layers to enhance connectivity. The FCL learns the dependencies and relationships in EEG data. In the FCL, a linear transformation is applied to the input vector via FCL weights. Later, the non-linear activation function is applied to the product, as given in Equation (30).

(30) $$y_{jk}\left(x\right)=f\left(\sum_{i=1}^{nH}W_{jk}x_{i}+W_{jo}\right)$$

where x represents the input flattened vector to the FCL, $W_{0}$ is the bias, and f is the non-linear activation function. The Softmax classifier computes the probability of the output class using Equation (31). The class label with the highest probability is chosen as the output class, as given in Equation (32). The SML activation function is given by the equation. $Z_{i}$ indicates the output layer value, which is given by Equation (33): $P_{i}$ is the probability of the output class level, and Ŷ indicates the predicted class label.

(31) $$P_{i}=softmax{\left(z\right)}_{i}=\frac{e^{z_{i}}}{\sum_{j=1}^{n}e^{z_{i}}}$$

(32) $$Ŷ=\underset{i}{\max}\left(P_{i}\right)$$

(33) $$Z_{i}=\sum_{j}^{i}h_{j}\cdot W_{ji}$$

The mini-batch gradient descent algorithm (MBGDM), which combines batch gradient descent (BGD) and stochastic gradient descent (SGD) to achieve lower computational efforts and robustness, is utilized for training the DL frameworks. MBGDM splits the training data into fewer batches (b) to reduce training time. The weights of the DL framework are modified using the error function described in the equation.

(34) $$E_{t}\left[f(w)\right]=\frac{1}{b}\sum_{i=\left(t-1\right)b+1}^{tb}f(w,x_{i})$$

where *x_i_* is the *i*th feature set of the training data, and MBGDM considers the initial learning rate *μ* = 0.001 to modify the weights, as described in Equation (35).

(35) $$W^{t+1}=W^{t}-\mu \nabla_{w}E\left[f(w^{t})\right]$$

Here, $W^{t+1}$ denoted modified weights, $W^{t}$ signifies older weights, $E\left[f(w^{t})\right]$ describes the error function and $\nabla_{w}$ symbolizes the gradient.

**B.**  ***Bi-LSTM***

Bi-LSTM provides a temporal representation of EEG features and long-range connectivity in the local and global features of the EEG. The proposed scheme uses two Bi-LSTM layers with 50 hidden layers to represent the features [33,34].

BiLSTM is an extension of the LSTM, which provides forward and backward long-term dependencies in the EEG. Understanding the context of the sequence in both directions is essential for time series stress analysis. In the input sequence from *t* = 1 to *t* = *T*, the BiLSTM incorporates a forward LSTM model to learn forward dependence. The input sequence from *t* = *T* to *t* = 1 incorporates a backward LSTM model to learn backward reliance. After flattening the PDCNN’s output, the BiLSTM determined the forward and backward states for the input sequence {x_1,x_2,…,x_T} using Equations (36) and (37), respectively.

(36) $${\bar{h}}_{t}=LSTM_{forward}\left(x_{t},{\bar{h}}_{t-1}\right)$$

(37) $${\underline{h}}_{t}=LSTM_{backward}\left(x_{t},{\underline{h}}_{t+1}\right)$$

The output of the BiLSTM combines the backward and forward hidden states at time *t*, as given in Equation (38). The symbol $h_{t}$ represents the final hidden state of the BiLSTM at time *t*, and the operator represents the concatenation of the backward and forward states.

(38) $$h_{t}=\left[{\bar{h}}_{t};\;{\underline{h}}_{t}\right]$$

The LSTM provides temporal portrayal and long-term connection in complex stress aspects. It is responsible for regulating the flow of information inside the model and comprises the forget gate, the input gate, and the output gate. The information stored in the cell state is discarded via the forget gate. A representation of the forgetting gate may be seen in Equation (39). Several symbols are used in this context: $x_{t}$ represents the input state at time step $t,h_{t-1}$ represents the hidden state from the previous step, $W_{f}$ represents the weight matrix of the forget gate, $b_{f}$ represents the bias value, and σ represents the sigmoid activation function.

(39) $$f_{t}=\sigma \left(W_{f}\cdot \left[h_{t-1},x_{t}\right]+b_{f}\right)$$

The input gate $\left(i_{t}\right)$ and candidate values $\left({\tilde{C}}_{t}\right)$ add the new information in the cell state as given in Equations (40) and (41), respectively. The cell state update $\left(C_{t}\right)$ combines the new information and the forgot gate’s output as given in Equation (42). The output gate $o_{t}$ produces the final output considering the hidden state $h_{t}$ as given in Equations (43) and (44), respectively.

(40) $$i_{t}=\sigma \left(W_{i}\cdot \left[h_{t-1},x_{t}\right]+b_{i}\right)$$

(41) $${\tilde{C}}_{t}=\tanh\left(W_{C}\cdot \left[h_{t-1},x_{t}\right]+b_{C}\right)$$

(42) $$C_{t}=f_{t}⊙C_{t-1}+i_{t}⊙{\tilde{C}}_{t}$$

(43) $$o_{t}=\sigma \left(W_{o}\cdot \left[h_{t-1},x_{t}\right]+b_{o}\right)$$

(44) $$h_{t}=o_{t}⊙\tanh\left(C_{t}\right)$$

**C.**  ***Deep Belief Network***

The DBN provides multilevel hierarchical features using multiple restricted Boltzmann Machines (RBMs). RBMs act as hidden layers and learn the connectivity and correlation between the input and hidden layers. The RBM attempts to minimize the energy required to learn the fundamental probability distribution of the EEG features as given in Equation (45) [35,36].

(45) $$E\left(v,h\right)=-\sum_{i=1}^{V}v_{i}b_{i}-\sum_{j=1}^{H}h_{j}c_{j}-\sum_{i=1}^{V}\sum_{j=1}^{H}v_{i}h_{j}W_{ij}$$

The RBM computes the energy for every configuration of the input (visible) and hidden layers using Equation. Here, $v_{i}$ denotes a binary state of the input layer, $h_{j}$ signifies a binary state of the hidden layer, $b_{i}$ stands for bias values of the input layer, $h_{j}$ symbolizes bias values of the hidden layer, V denotes the number of input (visible) layers, H stands for the number of hidden layers, and $W_{ij}$ denotes weights linking the input and hidden layers. The RBM assigns probability *p*(*v*) to the visible vector *v* using Equation (46).

(46) $$p\left(v\right)=\frac{\sum_{h}e^{-E\left(v,h\right)}}{\sum_{u}\sum_{h}e^{-E\left(u,h\right)}}$$

As the connection between the hidden and hidden layers is unavailable, the conditional distribution *p*(*h*|*v*) is factorial and is depicted by Equation (47).

(47) $$p\left(h_{j}=1|v\right)=\sigma (a_{j}+\sum_{i=1}^{V}w_{ij}v_{i})$$

Similarly, the connection between the visible and invisible layers was unavailable. The conditional distribution *p*(*v*|*h*) is factorial and depicted by Equation (48).

(48) $$p\left(v_{j}=1|h\right)=\sigma (b_{j}+\sum_{j=1}^{H}w_{ij}h_{j})$$

The $\sigma \left(x\right)$ indicates the sigmoid function and is denoted by Equation (49).

(49) $$\sigma \left(x\right)=\frac{1}{\left(1+e^{-x}\right)}$$

The features of the last layer of the DCNN, BilSTM, and DBN are concatenated together and provided to the FC layer for connectivity improvement. Finally, a softmax classifier is utilized for the classification of the stress. The BDDNet is optimized for hyper-parameter tuning using novel EOA.

## 5. EOA for Hyper-Parameter Optimization

Employee satisfaction is crucial in private and government sectors to achieve the highest throughput of the employee for generating maximum profit and fulfilling employee satisfaction and personal needs. The novel EOA is motivated by the employee appraisal process in organizations where the employees are motivated for good work, penalized or warned for mistakes, and trained or mentored to enhance their professional skills. Here, the EOA is utilized for the hyper-parameter tuning of the BDDNet, such as learning rate, decay rate, and momentum, which are usually manually optimized and lead to poor performance for hybrid DL frameworks.

In EOA, the initial population of N employee is created for the n employee variables representing the performance variables. Here, *N* is analogous to the total possible solutions, and n denotes problem variables. The initial population of the employees is set randomly in 0 to 1 using Equation (50) where $EM_{1},EM_{2},\ldots EM_{N}$ represent the employees of the organization.


(50) $$EM=\left[\begin{array}{c} EM_{1}\\ EM_{2}\\ \vdots \\ EM_{N} \end{array}\right]\left[\begin{array}{cccc} {em}_{11} & em_{12} & \cdots & em_{1n}\\ em_{21} & em_{22} & \ldots & em_{2n}\\ \vdots & \vdots & \ddots & \vdots \\ em_{N1} & em_{N2} & \cdots & em\_Nn \end{array}\right]$$

The fitness of each employee is considered based on the training error of the BDDNet. The employees providing better fitness for the iteration are directly passed to the next iterations as the reward policy, and other employees are trained or mentored based on exploration and exploitation and exploration strategy. During exploration, the employees are trained by providing the external training using Equation (51), and during exploitation, the employees are optimized based on mentoring from the best employees ($EM_{Best}$) of the organization using Equation (52).

(51) $$EM_{i}^{new}=EM_{i}+r_{1}*\left(EM_{i}-EM_{rand}\right)$$

(52) $$EM_{i}^{new}=EM_{i}+r_{2}*\left(EM_{i}-EM_{Best}\right)$$

Here, $EM_{i}^{new}$ is the updated population, and $r_{1}$ and $r_{2}$ are the arbitrary numbers in 0 to 1. The population is updated to 100 iterations, and the final optimized solution, having a lower error rate, is considered for training the BDDNet.

## 6. Experimental Results and Discussions

This section provides discussions on the experimental results carried out for the stress detection on the DEAP dataset.

### 6.1. Simulation System and Parameter Configurations

The proposed BDDNet was trained using the MBGDM algorithm for 200 epochs with an initial learning rate of 0.001 and a cross-entropy loss function. The dataset was split at a ratio of 70:30 for training and testing. The BDDNet achieved a training accuracy of 100% for the 200 epochs. The system parameter configurations are presented in Table 3.

### 6.2. Dataset: DEAP

The DEAP dataset consists of EEG samples of 32 subjects recorded while watching musical videos [37]. The participants rated the videos based on valence, arousal, dislike, like, familiarity, and dominance. The EEGs were down-sampled to 128 Hz and segmented to 60 s duration. The dataset consists of 40 trials with 40 channels. Each EEG channel consists of 8064 samples. The samples included 32 EEG signals, two EOGs (horizontal and vertical), Zygomaticus EMG, trapezius EMG, GSR, temperature, respiration belt, and plethysmograph. A self-assessment manikin (SAM) scale based on Russell’s paradigm for emotion analysis was used to quantify valence (*val*) and arousal (*arl*). While low arousal and high valences are regarded as calm, high arousal and low valence are considered tension. The arousal is considered as the variations in amplitudes, and valence is considered as variations in the temporal properties of the EEG. The arousal and valence values are in the range of 0 to 9 obtained using the SAM method, where lower arousal (*arl* < 4) and higher valence (4 < *val* < 6) depict the positive and relaxed state. The higher arousal level (*arl* > 5) and lower valence (*val* < 3) describe negative emotion and high tension [37,38,39]. The Equations (53) and (54) explain how arousal and valence levels determine whether an EEG signal is calm or anxious, respectively. This analysis yielded 140 stress signals and 104 calm signals.

(53) Calm=(arl<4)∩(4<val<6)

(54) Stress=(arl>5)∩(val<3)

### 6.3. Performance Metrics

The outcomes of the proposed stress detection scheme were analyzed using different evaluation metrics. Recall and precision offer quantitative and qualitative measures of stress detection. The F1-score provides the balance in recall and precision, which depicts the balance in the accuracy of the two classes. The selectivity and negative predictive rate (NPV) were used to measure the absence of stress. The selectivity ensures the model’s ability, and NPV evaluates the reliability for predicting the calm state correctly. Equations (55)–(60) provide different evaluation metrics, where *TPn* represents the true-positive value, *TNn* denotes the true-negative value, *FPn* signifies the false-positive value, and *FNn* denotes the false-negative value for stress detection.

(55) $$recall=\frac{TPn}{TPn+FNn}$$

(56) $$precision=\frac{TPn}{TPn+FPn}$$

(57) $$F1\text{-}score=\frac{2*recall*precision}{recall+precision}$$

(58) $$Selectivity=\frac{TNn}{TNn+FPn}$$

(59) $$NPV=\frac{TNn}{TNn+FNn}$$

(60) $$Accuracy=\frac{TPn+TNn}{TPn+TNn+FNn+FPn}$$

### 6.4. Discussions on Results for DEAP Dataset

**Figure 3:**
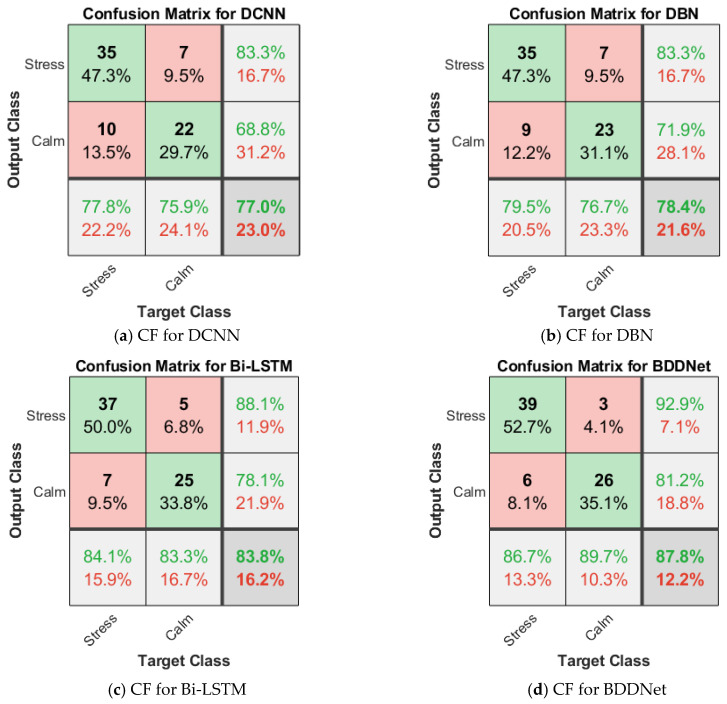
Confusion matrix for 2-class stress detection for BDDNet (40 channels).

**Figure 4:**
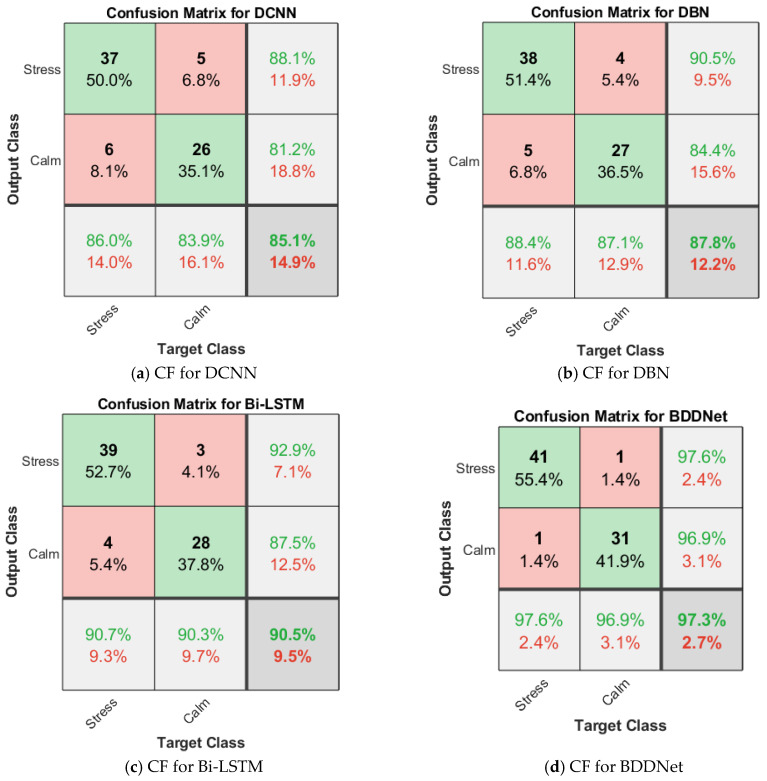
Confusion matrix for 2-class stress detection for BDDNet (15 channels).

Figure 3 and Figure 4 show the confusion matrix (CF) for the different classifiers for the two-class stress detection for the BDDNet with all 40 channels and BDDNet-ICA with five channels, respectively.

The overall results of the proposed stress-detection scheme for different DL frameworks are presented in Table 4. The proposed BDDNet provides superior results compared to traditional classifiers. The BDDNet-ICSA provides a superior accuracy of 97.3% compared to Bi-LSTM (90.5%), DBN (87.8%), and DCNN (85.1%) for five channels selected using ICSA. ICA chooses prominent channels with higher information, lower intra-class variability, and higher inter-class variability. It offers improved results compared to the results for the original dataset. The hybrid BDDNet-ICSA provides better spectral–temporal representation, long-term dependency, and multilevel hierarchical features, and assists in achieving improvements of 14.33%, 10.82%, and 7.51% in accuracy over the DCNN, DBN, and Bi-LSTM, respectively. The BDDNet provides disparity in qualitative measures (precision of 86.7%) and a quantitative measure (recall of 92.9%) for the 40 channels because of redundant and non-salient information in the EEG. The BDDNet without channel selection resulted in poor selectivity (81.2%) and lower accuracy (87.8%) for the hybrid BDDNet.

**Figure 5:**
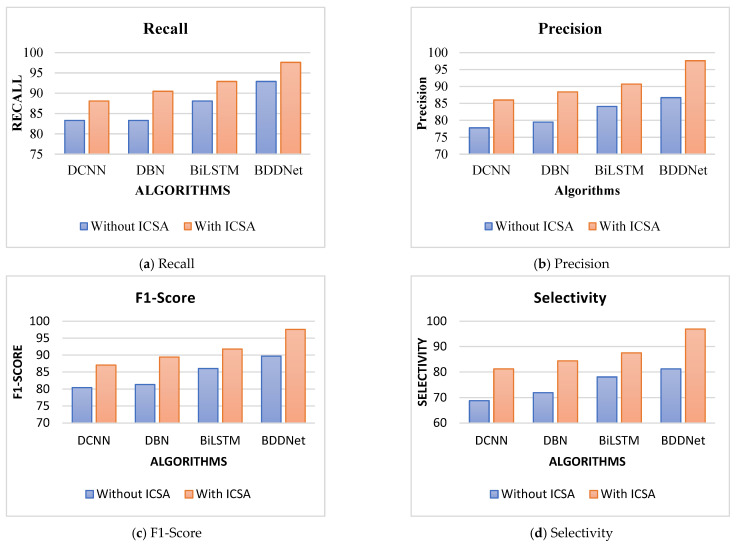

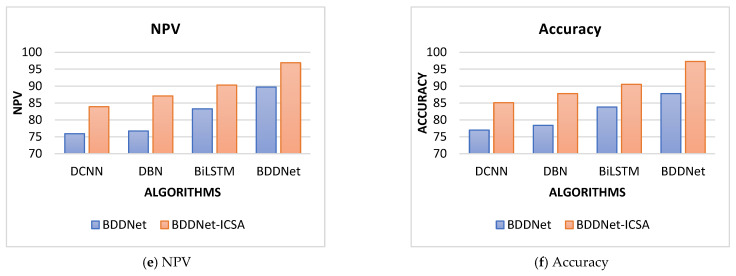
Visualizations of the results of stress detection scheme for DEAP.

However, channel selection using ICSA helps stabilize the training process and improves the training accuracy, as given in Figure 5. The BDDNet-ICSA provides a good balance between recall (97.6%) and precision (97.6%). BDDNet-ICSA provided an improved F1-score of 97.6% compared with the F1-score achieved for the BDDNet (89.69%). The BDDNet provides a recall of 92.9%, a precision of 86.7%, an F1-score of 89.69%, a selectivity of 81.2%, an NPV of 89.7%, and an accuracy of 87.8% for the 40-channel EEG data. However, the BDDNet-ICA provides enhanced recall of 97.6%, precision of 97.6%, F1-score of 97.6%, selectivity of 96.9%, NPV of 96.9%, and accuracy of 97.3% for the 15-channel EEG.

**Figure 6:**
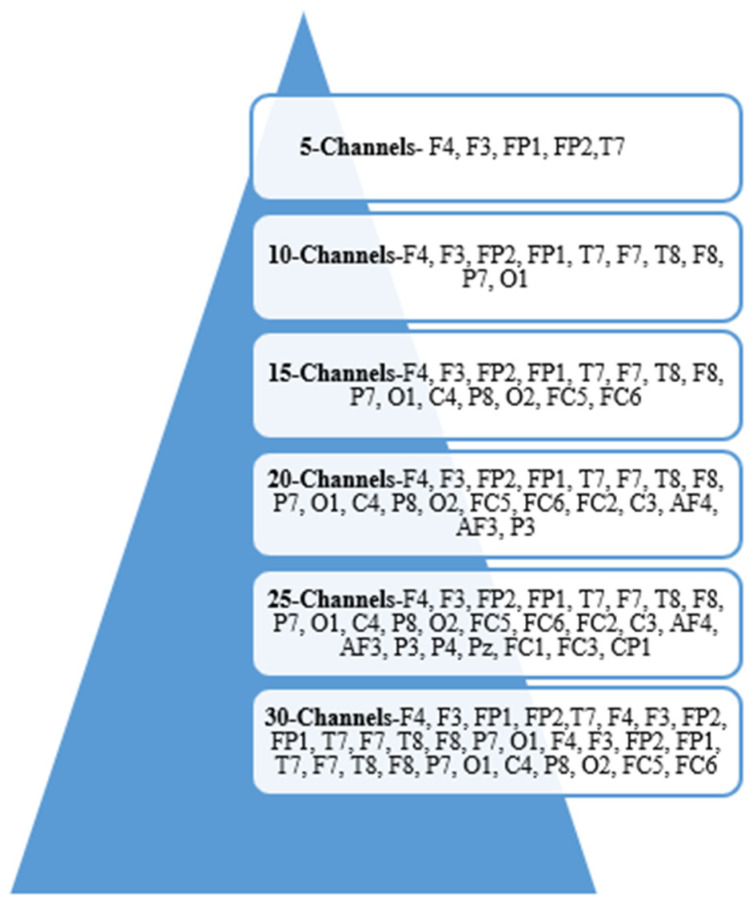
Channels selected using ICSA for DEAP.

The salient channels selected using ICSA are visualized in Figure 6. The ICSA provides the F4, F3, FP1, FP2, and T7 channels when 5-channels are selected: F4, F3, FP2, FP1, T7, F7, T8, F8, P7, and O1 when 10 channels are selected, and F4, F3, FP2, FP1, T7, F7, T8, F8, P7, O1, C4, P8, O2, FC5, FC6, FC2, C3, AF4, AF3, and P3 when 20-channels are chosen using SMO. The selected EEG channels are crucial for stress detection, covering the brain regions involved in emotional processing, cognitive workload, and autonomic responses. Frontal channels (F3, F4, FP1, FP2, AF3, AF4, F7, and F8) capture stress-related asymmetry in the prefrontal cortex, where the right side is more active during stress. Temporal channels (T7, T8, F7, and F8) are linked to the amygdala and regulate fear and emotional reactions. The central (C3, C4, FC2, FC5, and FC6) and parietal (P3, P7, and P8) channels reflect cognitive stress, attention modulation, and sensorimotor responses. Occipital channels (O1 and O2) help monitor stress-induced changes in visual perception, particularly through alpha wave suppression.

**Figure 7:**
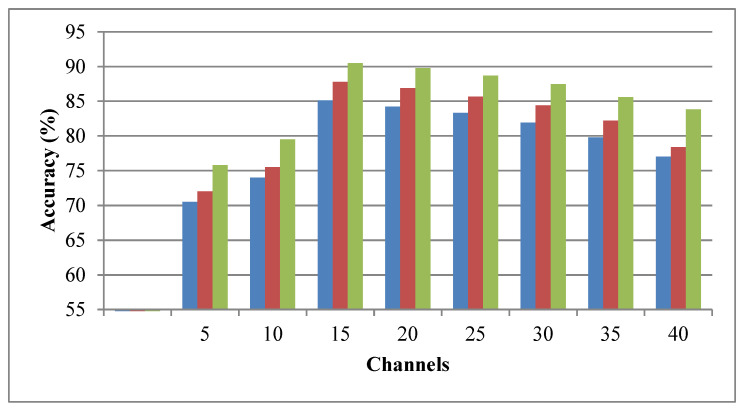
Accuracy for different channel selections using ICSA for BDDNet.

Table 5 and Figure 7 offer the comparative analysis of BDDNet-based stress detection for different channels selected using ICSA. At 5 channels, BDDNet achieves 80.4%, improving to 84.2% at 10 channels and reaching a maximum of 97.7% at 15 channels. Similar trends are seen in other models. Beyond 15 channels, performance declines slightly; BDDNet drops to 96.8% (20 channels) and 87.8% (40 channels) due to redundancy in features. We have chosen 15 channels for final implementation, which leads to better accuracy and lower computational intricacy.

### 6.5. Discussions on Results for SEED Dataset

We have also evaluated the effectiveness of the proposed dataset for the SEED dataset, which consists of a total of 62 channels [40], to analyze the generalization capability of the proposed system. The effectiveness of the system is evaluated for the SEED dataset for ICSA-based channel selection and without channel selection, as given in Table 6. The BDDNet provides better results for channel selection using ICSA for the 20 channels. The BDDNet without channel selection resulted in an overall accuracy of 82.51%, a recall of 88.32%, a precision of 83.09%, an F1-score of 85.62%, an NPV of 86.43%, and a selectivity of 76.21% for 62 channels. The BDDNet with ICSA achieves an overall accuracy of 92.63%, a recall of 92.79%, a precision of 92.52%, an F1-score of 92.66%, a specificity of 91.48%, and an NPV of 91.68%, demonstrating a notable improvement over the traditional technique. With channel selection, the system achieves overall accuracies of 82.06% for DCNN, 82.59% for DBN, 86.97% for BiLSTM, and 92.62% for BDDNet, optimized using EOA for 20 channels. The proposed algorithm yields superior results for the SEED dataset, also demonstrating generalization capability for a stress detection system.

On the SEED dataset, BDDNet outperforms DCNN, DBN, and Bi-LSTM across almost all EEG channel configurations as given in Table 7. Its accuracy improves steadily with an increase in channels, reaching a peak of 92.62% with 20 channels, significantly higher than Bi-LSTM (86.97%), DBN (82.59%), and DCNN (82.06%). Even with 25 and 30 channels, BDDNet maintains strong performance (91.80% and 91.50%), demonstrating its ability to leverage richer feature sets effectively. Although accuracy slightly drops beyond 35 channels due to redundant information, BDDNet continues to lead, achieving 77.50% with 62 channels, demonstrating its superior feature learning and robustness for stress detection.

### 6.6. Discussions on Results for Different Channel Selection Techniques

Table 8 highlights the role of channel selection techniques in improving stress detection performance using the BDDNet-EOA model on the DEAP and SEED datasets. The optimal selection of EEG channels reduces redundant information, focuses on the most discriminative features, and thereby improves classification accuracy. Among the techniques, Genetic Algorithm (GA) and Particle Swarm Optimization (PSO) show significant gains, with PSO achieving 95.25% on DEAP and 88.2% on SEED. However, the CSA outperforms GA and PSO, achieving 96.8% (DEAP) and 90.25% (SEED) due to its balanced exploration and exploitation strategy, which prevents premature convergence. CSA is inspired by the intelligent behavior of crows in hiding and retrieving food, enabling it to dynamically switch between global and local searches. Unlike GA and PSO, which can become trapped in local optima, CSA effectively maintains diversity in the search space, resulting in better channel subset selection and higher accuracy. When further enhanced with Improved CSA (ICSA), the performance peaks at 97.3% (DEAP) and 92.62% (SEED), making it the most effective approach for stress detection among all compared techniques.

### 6.7. Discussions on Comparative Analysis of Results with Traditional Techniques

A comparison of the proposed scheme with the traditional state of the art is provided in Table 7. Li et al. [23] presented inter-frequency band mapping (IFBM) features for depicting the distinctiveness of EEGs for stress analysis. It provides 95.15% accuracy for spatial–frequency convolutional self-attention networks (SFCSAN). Kim et al. [24] suggested a 3-D convolutional gated self-attention DNN (3DCGSA) along with IBFM for stress detection, which showed 96.68% accuracy for stress detection. Saranya and Jayanthy [22] explored the affinity propagation. This artificial neural network achieved an overall accuracy of 86.80% for two-class stress detection, utilizing an experimental selection strategy for channel selection. The feature representation using a time-domain wavelet-based time–frequency domain depiction of EEG, presented by Hasan and Kim [39], offers an overall accuracy of 73.8% for the KNN classifier. The CBGG presented by Roy et al. [11] provides an overall accuracy of 96.35% for the DEAP dataset. The 1-D DCNN and LSTM achieve an overall accuracy of 88.03% for the DEAP dataset, which effectively captures temporal depiction using LSTM but lacks generalization capability (Table 9).

The proposed EOA-optimized BDDNet-ICSA offers an improved accuracy of 97.3% for 5-channel EEGs. The preprocessing of the EECG using WPT-based soft thresholding helps minimize the noise and artifacts in the EEG signal. The WPT helps retain the structural content of the EEG and demonstrates superior accuracy for 87.8% of the 40 EEG channels and 97.3% for the five channels selected using ICSA for stress detection. However, without EEG filtering, the BDDNet provides 82.25% and 85.45% accuracy for 40 channels and 15 channels, respectively.

## 7. Conclusions

Thus, this article presents stress detection using a novel hybrid BDDNet that combines a DCNN, BiLSTM, and DBN. It helps improve feature distinctiveness, spectral–temporal depiction, long-term dependency, and multilevel abstracted hierarchical features. The competitive improved CSA provides efficient channel selection, offering several benefits, including the capacity to self-organize, simplicity, flexibility, robustness, and scalability. The novel EOA is used to optimize the hyper-parameters of the BDDNet, such as learning rate, decay rate, and momentum. The performance of the EOA-optimized BDDNet-ICSA was evaluated on the DEAP dataset, yielding enhanced recall, precision, F1-score, selectivity, NPV, and accuracy of 97.6%, 97.6%, 97.6%, 96.9%, 96.9%, and 97.3%, respectively, for the 15-channel EEG. The proposed BDDNet offers an overall accuracy of 92.62% for the SEED dataset and helps to validate the generalization capability of the system. The complexity in the DL framework may limit the deployment flexibility of the suggested system on the resource-constrained standalone devices. As DL architectures are highly abstracted, the interpretability and explainability of the stress detection system are inferior, which limits the acquisition of trust and reliability in real-time critical applications. The disparity in the calm and stress samples leads to a class imbalance problem. In the future, the focus should be on improving the interpretability and explainability of the system. In the future, the effectiveness of the stress detection scheme can be improved by generating synthetic samples using data augmentation to lessen the class imbalance issue. Additionally, the effectiveness of the system can be enhanced by implementing an efficient feature selection scheme to minimize the computational complexity of the stress detection framework.

## Figures and Tables

**Table 1:** EEG Signal details.

EEG Band	Amplitude	Frequency	Mental State
Delta (δ)	100–200 µv	0.5–4 Hz	Brain injury, deep sleep, unconsciousness
Theta (θ)	20–100 µv	4–8 Hz	Meditation, drowsiness, creativity
Alpha (α)	20–60 µv	8–13 Hz	Calm, relaxed, awake (not alert)
Beta (β)	5–20 µv	13–30 Hz	Problem-solving, alert, active thinking
Gamma (γ)	3–10 µv	30–100 Hz	Perception, attention, high-level cognition

**Table 2:** Details of EEG Features.

Types of Features	Features	Total Features
Time-domain Features	Mean	1
	Standard Deviation	1
	Variance	1
	Median	1
	Skewness	1
	ZCR	1
	Activity	1
	Mobility	1
	Complexity	1
	RMS	1
	Shannon Entropy	1
	Line Length	1
	Non-linear Energy	1
Frequency-Domain Features	WPT	224
	Energy	1
	IWMF	1
	IWBF	1
	Spectral Kurtosis	257
Textural Features	LBP	10
	LNDP	10
	LGP	10
Total Features		527

**Table 3:** Initial parameter configurations of the BDDNet.

Parameter	Specification
Learning Algorithm	MBGDM
Initial learning rate	0.001
Loss function	Cross-entropy
Epoch	200
Dropout	0.5
Training Testing ratio	70:30
DCNN Filters	First layer-64, Second Layer-128, Third layer-256
DCNN Filter Size	3 × 1
Hidden Units in DBN Layers	RBM 1—200, RBM 2—150, RBM 3—100
BiLSTM Layers	2 Layers (50 Gates)

**Table 4:** Comparative Results for the Stress Detection for Different DL Frameworks for DEAP.

Performance Metrics	Without Channel Selection (40 Channels)				With Channel Selection (15 Channels)			
	DCNN	DBN	Bi-LSTM	BDDNet	DCNN	DBN	Bi-LSTM	BDDNet
**Recall**	83.3	83.3	88.1	92.9	88.1	90.5	92.9	97.6
**Precision**	77.8	79.5	84.1	86.7	86	88.4	90.7	97.6
**F1-Score**	80.46	81.36	86.05	89.69	87.04	89.44	91.79	97.6
**Selectivity**	68.8	71.9	78.1	81.2	81.2	84.4	87.5	96.9
**NPV**	75.9	76.7	83.3	89.7	83.9	87.1	90.3	96.9
**Accuracy**	77	78.4	83.8	87.8	85.1	87.8	90.5	97.7

**Table 5:** Comparative Analysis of BDDNet-Based Stress Detection for Different Channels for DEAP.

Number of Channels Selected	Accuracy (%)			
	DCNN	DBN	Bi-LSTM	BDDNet
**5**	70.5	72.0	75.8	80.4
**10**	74.0	75.5	79.5	84.2
**15**	85.1	87.8	90.5	97.7
**20**	84.2	86.9	89.8	96.8
**25**	83.3	85.7	88.7	95.9
**30**	81.9	84.4	87.5	94.5
**35**	79.8	82.2	85.6	92.6
**40**	77.0	78.4	83.8	87.8

**Table 6:** Comparative Results for the Stress Detection for Different DL Frameworks for SEED.

Performance Metrics	Without Channel Selection (62 Channels)				With Channel Selection (20 Channels)			
	DCNN	DBN	Bi-LSTM	BDDNet	DCNN	DBN	Bi-LSTM	BDDNet
**Recall**	79.40	77.61	83.57	88.32	84.97	86.18	87.34	92.79
**Precision**	73.14	76.41	79.59	83.09	80.69	83.64	84.98	92.53
**F1-Score**	76.14	77.01	81.53	85.62	82.78	84.89	86.14	92.66
**Selectivity**	63.13	66.27	73.01	76.21	78.16	81.13	82.15	91.48
**NPV**	70.96	70.78	78.86	86.43	80.35	81.58	86.84	91.68
**Accuracy**	73.65	74.93	79.87	82.51	82.06	82.59	86.97	92.62

**Table 7:** Comparative Analysis of BDDNet-Based Stress Detection for Different Channels for SEED.

Number of Channels Selected	Accuracy (%)			
	DCNN	DBN	Bi-LSTM	BDDNet
**5**	70.50	72.00	75.80	80.40
**10**	72.25	73.75	77.65	82.30
**15**	79.55	81.65	85.00	90.95
**20**	**82.06**	**82.59**	**86.97**	**92.62**
**25**	81.90	84.40	86.50	91.80
**30**	80.85	83.30	86.05	91.50
**35**	79.80	82.20	85.60	88.80
**40**	78.40	80.30	84.70	85.45
**45**	77.00	78.40	83.80	84.20
**50**	76.45	78.05	82.56	82.65
**55**	76.18	77.88	81.94	79.85
**62**	75.90	77.70	81.33	77.50

**Table 8:** The role of channel selection techniques in improving stress detection performance using the BDDNet-EOA model on the DEAP and SEED datasets.

Stress Detection Method	Channel Selection	DEAP (15 Channels)	SEED (20 Channels)
BDDNet-EOA	GA	93.25	87.25
BDDNet-EOA	PSO	95.25	88.20
BDDNet-EOA	CSA	96.80	90.25
BDDNet-EOA	ICSA	97.30	92.62

**Table 9:** Comparative Analysis with Traditional State of the Art for DEAP Dataset.

Author	Pre-Processing	Channel Selection	Feature Representation	Method	Accuracy
Li et al. (2022) [23]	-	-	IFBM	SFCSAN	95.15%
Kim et al. (2022) [24]	-	-	IFBM	3DCGSA	96.68%
Saranya and Jayanthy (2022) [22]	-	Experimental selection	Multiple spectral and statistical features Pearson correlation coefficient (PCC) for feature selection	AP-ANN	86.80%
Hasan and Kim (2019) [39]	Band Pass Filter	-	Time-domain and wavelet time-frequency features	KNN	73.38%
Roy et al. [11]	Band Pass Filter	-	DWT	CBGG	96.35%
Patel et al. [13]	Band Pass Filter	-	-	1-D CNN and BiLSTM	88.03%
Proposed Method	-	-	MEG	BDDNet	82.25%
	-	ICSA	MEG	BDDNet	85.45%
	WPT	-	MEG	BDDNet	87.8%
	WPT	ICSA	MEG	BDDNet	97.3%

## Data Availability

The DEAP dataset is publically available at https://www.eecs.qmul.ac.uk/mmv/datasets/deap/ accessed on 27 July 2025.
